# Successful Multidisciplinary Treatment, Including Atezolizumab Plus Bevacizumab Biological Therapy, for Multiple Hepatocellular Carcinomas Adjacent to Major Vessels: A Case Report

**DOI:** 10.7759/cureus.53997

**Published:** 2024-02-11

**Authors:** Kei Harada, Takahisa Fujikawa, Yusuke Uemoto, Yuki Aibe

**Affiliations:** 1 Surgery, Kokura Memorial Hospital, Kitakyushu, JPN; 2 Gastroenterology, Kokura Memorial Hospital, Kitakyushu, JPN

**Keywords:** radiofrequency ablation, laparoscopic liver resection, multidisciplinary treatment, multiple hepatocellular carcinomas, atezolizumab plus bevacizumab

## Abstract

Multiple hepatocellular carcinomas (HCCs) are currently being treated with multimodal therapy that includes liver resection and local therapy. Although the necessity of multimodal therapy for multiple HCCs is evident, treating them is extremely difficult due to the complex nature of multiple HCCs and the frequent occurrence of underlying liver damage. We encountered a case in which long-term tumor control was achieved through multidisciplinary treatment, including atezolizumab plus bevacizumab combination biological therapy. As in the current case, less-invasive surgical resection combined with radiofrequency ablation after a combination of biological therapy may be one of the preferred options for the treatment of initially unresectable multiple HCCs.

## Introduction

Hepatocellular carcinoma (HCC) is a common cause of death connected to cancer, and there is a growing incidence of HCC all over the world [1,2]. Especially with multiple HCCs, it is very complicated, and it is important to conduct multimodal therapy such as surgery, interventional radiology (IVR), radiofrequency ablation (RFA), and chemotherapy [3,4]. In recent years, significant progress has been made in the treatment of HCC, including the introduction of targeted agents and immune checkpoint inhibitors (ICIs) [5]. Specifically, the first-line treatment for unresectable HCC is now a combination of the programmed death-ligand 1 (PD-L1) monoclonal antibody atezolizumab and the anti-vascular endothelial growth factor (VEGF) receptor monoclonal antibody bevacizumab [6,7].

We experienced a case in which multiple HCCs adjacent to major vessels were successfully controlled by minimally invasive surgical resection combined with RFA after biologic therapy with atezolizumab and bevacizumab. It is anticipated that our case report will be used as a guide for future studies on multimodal therapy to usher in a hopeful new era in the management of multiple HCCs.

## Case presentation

**Figure 1:**
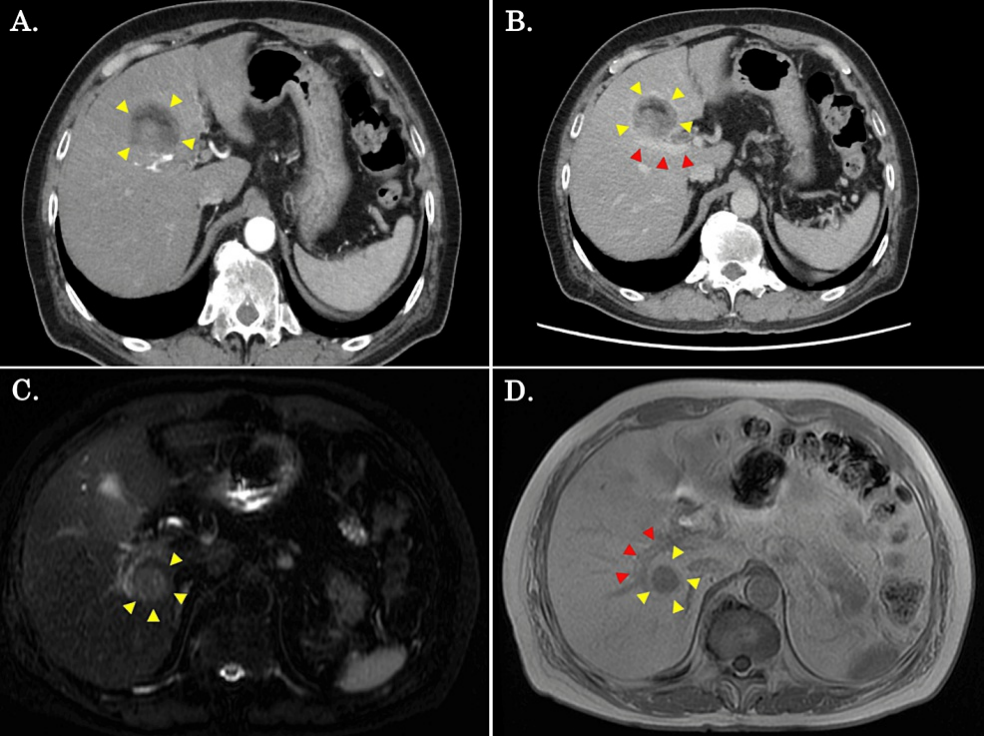
CT and MRI findings prior to the biological therapy. (A, B) An enhanced CT scan showed a 40 mm-sized S4/S5 tumor (yellow arrows), with broad contact with the Glissonean pedicle of the right anterior section (red arrows). (C, D) An MRI showed a 27 mm-sized S7 tumor (yellow arrows) adjacent to the right posterior Glisson (red arrows).

A 71-year-old man was referred to our hospital for the treatment of multiple HCCs. Although he did not have chronic liver disease due to the hepatitis B or C virus infection, he was a heavy drinker and suffered from alcoholic liver cirrhosis. Initial tumor marker levels were as follows: alpha-fetoprotein (AFP), 2.7 ng/mL; and protein induced by vitamin K absence or antagonist-II (PIVKA-II), 54.1 mAU/mL. An indocyanine green retention test at 15 min (ICG R15) was 35.6%. An enhanced computed tomography (CT) scan showed two liver lesions, including a 40 mm-sized S4/S5 tumor, with broad contact with the Glissonean pedicle of the right anterior section (Figures 1A-1B) and a 27 mm-sized S7 tumor adjacent to the right posterior Glisson (Figures 1C-1D). Although it was suggested that extended right hepatectomy was required for radical resection, we considered the current tumor unsuitable for resection due to his impaired liver functional status.

**Figure 2:**
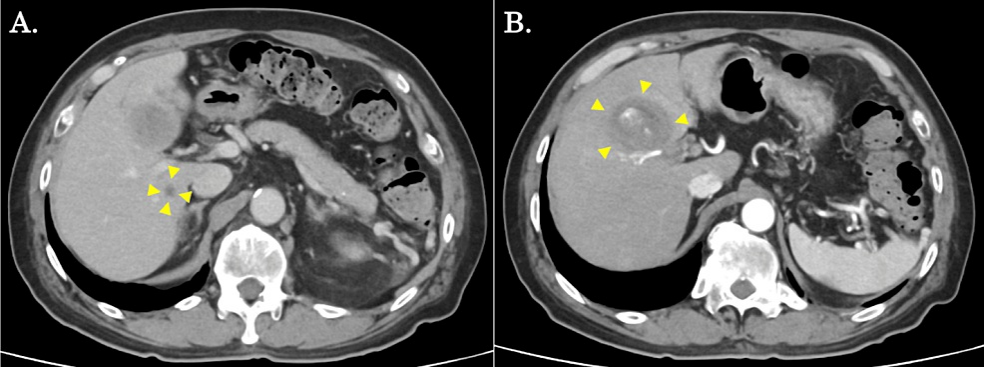
Contrast-enhanced CT findings after the biological therapy. (A) An axial contrast-enhanced CT scan revealed a reduction in the S7 tumor size of 9 mm (yellow arrows). (B) An axial contrast-enhanced CT scan revealed that the S4/5 tumor became slightly enlarged (47 mm in size) (yellow arrows).

The patient was immediately introduced to combination biological therapy with atezolizumab (1,200 mg) plus bevacizumab (10 mg/kg). After sobriety and six courses of biological therapy, his tumor marker levels did not change significantly, but his ICG R15 relatively improved from 35.6% to 24.1%. The follow-up CT scan showed that the S7 tumor was markedly decreased in size (9 mm in size) (Figure 2A), although the S4/S5 tumor became slightly enlarged (47 mm in size) (Figure 2B). At that point, laparoscopic extended medial sectionectomy with the preservation of the anterior Glisson for the S4/S5 tumor, combined with intraoperative RFA for the S7 tumor, was planned.

**Figure 3:**
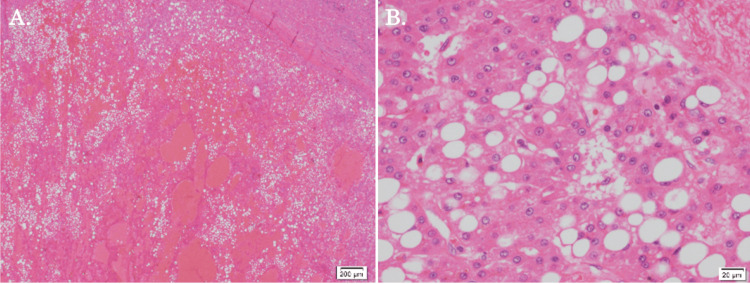
Pathological findings of the resected S4/S5 tumor. Hematoxylin and eosin staining of the tissues of the resected specimen revealed hepatocellular carcinoma, well-to-moderately differentiated, trabecular, and pseudoglandular with prominent fatty change and psoriasis. (A) Low-power-field view of the lesion (x40). (B) High-power-field view of the lesion (x400).

During the operation, we found that the clearly demarcated hepatic tumor was located adjacent to the anterior Glissonean pedicle but was able to be detached from it relatively easily. RFA for the S7 tumor, followed by curative-intent laparoscopic medial sectionectomy for the S4/S5 tumor, was successfully performed. The postoperative pathological examination of the S4/S5 tumor revealed well-to-moderately differentiated hepatocellular carcinoma with prominent fatty changes and psoriasis (Figure 3). The postoperative recovery went without incident, and he was discharged home on postoperative day seven.

**Figure 4:**
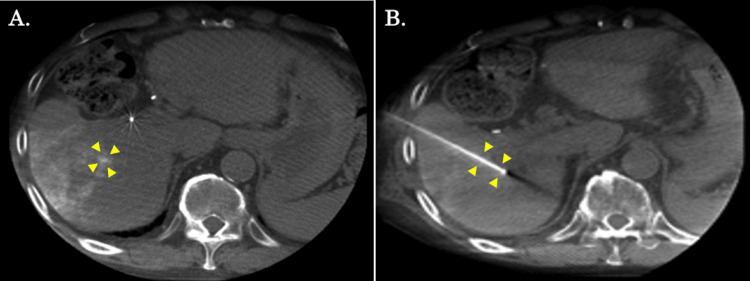
Cone beam CT findings during the radiofrequency ablation for the S8 tumor. (A) Cone beam CT shows lipiodol accumulation in the target lesion of liver S8 (yellow arrows). (B) Ablation is performed after the lesion has been pierced with a needle (yellow arrows).

**Figure 5:**
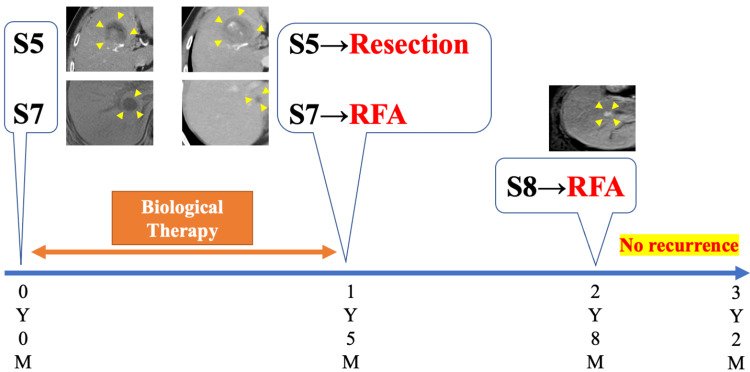
Clinical course of the current patient (the figure is the authors' own creation). RFA: radiofrequency ablation, Y: year, M: month, S: segment

The patient was doing well without recurrence 15 months after surgery and received no oncological therapy. However, during follow-up, a CT scan showed a recurrence of HCC at S8, and RFA was performed (Figure 4). The procedure was performed without any problems, and there has been no recurrence since then. The patient is still doing well and free from oncological therapy without any recurrence six months after the last intervention (three years and two months after the initial diagnosis). Figure 5 summarizes the clinical course of the current patient.

## Discussion

HCC is responsible for 80-90% of all primary liver malignancies, and liver cancer has the fourth-highest fatality rate in the world [8]. Additionally, multiple HCCs are associated with a worse prognosis than a single tumor, according to the findings of a number of studies [9]. It is recommended that the decision regarding treatment for multiple HCCs be based on a multidisciplinary interaction between several professionals. In addition, multimodal therapy with angiogenesis inhibitors, ICIs, chemotherapy, radiation, and surgery in an organic manner is clearly necessary for the treatment of multiple HCCs [10,11].

In a phase 1b study, patients with previously unresectable HCC who received atezolizumab plus bevacizumab combination chemotherapy demonstrated good anti-tumor activity, with a median progression-free survival (PFS) of 5.6 months, an overall response rate (ORR) of 27.3%, and a complete response (CR) of 5.5% [12]. In the current case, due to impaired liver function, two tumors, S4/S5 and S7, which have substantial contact with major Glissonean pedicles, were considered unresectable, and atezolizumab plus bevacizumab combination chemotherapy was started. We decided on surgery for S4/S5, which had enlarged somewhat after chemotherapy, and RFA for S7, which had significantly shrunk, following extensive consultation with several professionals.

Although surgical resection is the preferred treatment for HCC, RFA is indicated in cases of small HCC that cannot be resected, such as in our cases [13]. In addition, RFA has been the most commonly utilized technique for liver metastases from small malignant hepatic tumors, including HCC and colorectal cancer, because of its efficacy, safety, and generally favorable clinical results [14]. In fact, in our case, RFA was beneficial not just for S7, which had shrunk due to biological therapy, but also for a minor S8 lesion that had recurred.

With recent developments in surgical procedures and perioperative treatment, aggressive surgical resection for multiple HCCs has been recommended for patients who can efficiently excise all tumors and preserve liver function [15]. As in our case, just as minimally invasive liver resection using laparoscopy has helped maintain residual liver function and reduce the burden on patients, further advances in surgical procedures, represented by the spread of robotic surgery, will have a synergistic effect with the growth of other fields. It is believed that this will contribute to future multimodal therapy for multiple HCCs.

## Conclusions

We reported our experience with successful multidisciplinary treatment of the case with multiple HCCs adjacent to major vessels. As in the current case, less-invasive surgical resection combined with radiofrequency ablation after a combination of biological therapy may be one of the preferred options for the treatment of initially unresectable multiple HCCs. We hope that our case report may help future research into multimodal therapy for multiple HCCs.
